# Association Between Daily Mode of Transportation and Cognitive Function Among Older Adults With Mild Cognitive Impairment: A Cross-Sectional Observational Study

**DOI:** 10.7759/cureus.83021

**Published:** 2025-04-25

**Authors:** Teruaki Masuda, Emiko Segawa, Noriyuki Kimura, Takuma Sato, Kenichi Anabuki, Yoshitaka Nakamura, Shigenobu Mitsuzawa, Satoru Shinkawa, Ken Aoshima, Etsuro Matsubara

**Affiliations:** 1 Department of Neurology, Faculty of Medicine, Oita University, Yufu, JPN; 2 Human Biology Integration Foundation, Deep Human Biology Learning (DHBL), Eisai Co. Ltd Tsukuba Research Laboratories, Tsukuba, JPN; 3 Human Biology Integration Foundation, Deep Human Biology Learning (DHBL), Eisai Co. Ltd, Bunkyo-ku, JPN; 4 Innovative Research Excellence, Honda R &amp; D Co. Ltd, Wako, JPN; 5 Microbes &amp; Host Defense Domain, Deep Human Biology Learning (DHBL), Eisai Co. Ltd Tsukuba Research Laboratories, Tsukuba, JPN; 6 School of Integrative and Global Majors, University of Tsukuba, Tsukuba, JPN

**Keywords:** alzheimer’s disease, cognitive function, mild cognitive impairment, mode of transportation, preventive care

## Abstract

Background

Changes in mode of transportation, such as driving cessation, elevate the risk of mild cognitive impairment (MCI) and Alzheimer’s disease among older adults residing in communities. However, the association between transportation-related lifestyles and cognitive function in older adults with MCI remains unclear. This study aimed to explore the relationship between mobility independence and cognitive dysfunction among older patients with MCI.

Methods

This was a retrospective study of community-dwelling adults aged 65 years or older from Usuki, Oita Prefecture, Japan. Data from 117 participants with MCI were analyzed. Using the Lawton Instrumental Activities of Daily Living scale, participants were categorized into independent and dependent mobility groups based on their mode of transportation. Cognitive function was assessed using the Mini-Mental State Examination (MMSE) and the Japanese version of the Montreal Cognitive Assessment (MoCA-J), and scores were compared across modes of transportation.

Results

Significant differences were found in total MoCA-J scores, visuospatial executive function, naming, and orientation, and MMSE orientation of time and place, with higher scores observed in the independent mobility group compared to the dependent group (p < 0.05). Analysis of covariance further supported these findings, showing higher scores in total MoCA-J and MMSE scores in the independent mobility group.

Conclusions

This study highlights the relationship between daily mode of transportation and cognitive function in patients with MCI. Participants with independent mobility exhibited superior cognitive function compared to the dependent mobility group. These findings may contribute to the development of new interventions for preventing the transition from MCI to dementia by further validation of causal relationships in longitudinal or intervention studies.

## Introduction

Approximately 50 million people worldwide have dementia, and this number is expected to increase to 152 million by 2050 [1]. Dementia is a major global public health problem that imposes a severe burden on patients, caregivers, and society, and has considerable economic consequences as well [2]. Mild cognitive impairment (MCI) is a syndrome defined as a greater decline in cognitive function than would be expected for an individual’s age and educational level, with no particular impairment in activities of daily living (ADL) [3]. MCI is associated with an increased risk of dementia and 10-20% of patients diagnosed with MCI will eventually progress to develop AD in the future [3]. Various lifestyle interventions for MCI can reduce the progression of cognitive decline [4,5]. Therefore, understanding modifiable risk factors and developing evidence-based interventions to delay or prevent cognitive impairment is crucial.

A prior study proposed 12 modifiable risk factors for dementia: low education, hypertension, excessive alcohol consumption, hearing impairment, head injury, obesity, depression, lack of exercise, diabetes, social isolation, smoking, and air pollution [1]. Minimizing exposure and controlling these risk factors could prevent or delay the onset of 40% of dementia cases [1]. Mobility and car use are known lifestyle factors that affect the risk of dementia. Additionally, activities that require independent mobility, such as shopping and gardening, are known to help reduce the risk of dementia [6].

Although driving is often the preferred mode of transportation for older adults in Japan and other countries, it has become a societal issue due to traffic accidents involving older drivers. To encourage voluntary license surrender and prevent accidents, the National Police Agency of Japan mandates driving lessons, including on-road driving assessments and cognitive tests, for those aged 70 and 75 years renewing their licenses [7]. While patients with dementia are required to surrender their licenses, some patients with MCI continue to drive. For older adults, car ownership and driving are associated with independence and life satisfaction [8,9]. Driving cessation likely reduces outdoor activities and replaces them with indoor activities [10]. Although driving cessation increases the risk of functional limitations among older adults, the risk can be mitigated if independent mobility through public transportation is maintained [11]. Moreover, social isolation, a modifiable risk factor for dementia, is thought to result from dependent mobility, including the use of other modes of transportation. Previous studies have demonstrated the relationship between lifestyle factors related to mode of transportation and cognitive function among community-dwelling older adults [11-13]; however, this relationship has not been analyzed among older adults with MCI. Therefore, we aimed to explore the relationship between daily modes of transportation and cognitive dysfunction among older patients with MCI by examining data from participants in the MCI group of the USUKI cohort study [14,15].

## Materials and methods

This was a retrospective study involving the reanalysis of data obtained from the USUKI study [14,15]. 

The USUKI study

The USUKI clinical research study was a prospective cohort study conducted between 2015 and 2019 that examined the risks and protective lifestyle factors for dementia among older adults in Usuki, Oita Prefecture, Japan [14,15]. Adults aged 65 years or older account for approximately 38% of the Usuki population. Public servants recruited adults without dementia via public relations initiatives. The participants were 1,020 community-dwelling adults. The inclusion criteria were (i) being aged 65 years or older, (ii) living in Usuki, (iii) being physically and psychologically healthy, (iv) not diagnosed with all-cause dementia, and (v) having independent function in ADL. The exclusion criteria were (i) having a history of other neurological or psychiatric disorders, such as Parkinson’s disease and epilepsy, (ii) having a history of severe head trauma, alcoholism, severe cardiac failure, or severe hepatic or renal dysfunction, (iii) undergoing treatment for cancer, and (iv) experiencing difficulty walking.

The USUKI prospective cohort study was conducted in accordance with the Declaration of Helsinki [16] and was approved by the local ethics committee of Oita University Hospital (approval number: B15-006, UMIN No.: UMIN000017442). Written informed consent was obtained from all participants. The study followed the Strengthening the Reporting of Observational Studies in Epidemiology (STROBE) reporting guidelines.

Study Procedure

All participants underwent a physical examination, an evaluation of cognitive function, and a medical interview at baseline. Height and weight were measured, and body mass index was calculated as weight in kilograms divided by height in meters. We collected information on the participants’ demographic characteristics, namely, age, gender, education level, and smoking status, as well as their alcohol consumption and medication history. Information was obtained at baseline through interviews conducted by trained medical staff. History of chronic disease was defined as having a prior diagnosis of stroke, cardiac disease, hepatic disease, renal disease, or cancer. Assessment of vascular risk factors, such as hypertension, diabetes mellitus, and hypercholesterolemia, was based on a detailed clinical history and information regarding current medication, including antihypertensive, antidiabetic, and anticholesterol medication. Cognitive function was evaluated using the Mini-Mental State Examination (MMSE), a widely employed tool for dementia screening. Neurologists and clinical psychologists conducted primary screening for dementia using the MMSE results. Among the participants, seven had other neurological disorders, one had severe renal dysfunction, and four had difficulty walking without assistance. Moreover, 13 participants with dementia were identified based on interviews at the first examination. Thus, these 25 participants were excluded from the study. In addition, 140 participants with missing or inadequate data were excluded. Consequently, 855 participants were included in the study. 

Among the included 855 participants, 118 who were diagnosed with MCI underwent further assessments at baseline. The MCI diagnosis was made based on criteria presented in previous studies [14,15,17]: (i) subjective memory complaints and objective memory impairment, (ii) Clinical Dementia Rating score of 0.5, and (iii) absence of significant impairment in cognitive function and ADL. Trained medical staff collected data on life environment, clinical history, and medication using two types of self-reported questionnaires: the Lawton Instrumental ADL (IADL) scale and living environment and health behaviors. Cognitive assessments were performed using the Japanese version of the Montreal Cognitive Assessment (MoCA-J). Additionally, information on patient demographic characteristics, including age, sex, education duration, body mass index, smoking status, alcohol consumption, and medical history such as hypertension, diabetes, and hypercholesterolemia, was collected.

Participants for the present study

**Figure 1:**
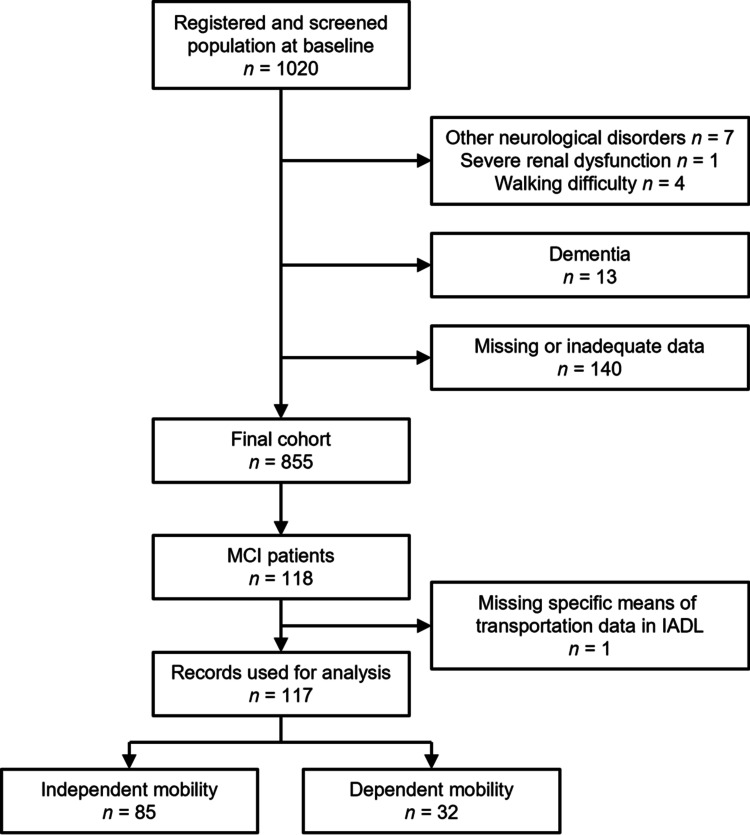
Selection flow of records for analysis. IADL, instrumental activities of daily living

To analyze the relationship between independence in daily mode of transportation and cognitive function among patients with MCI, we initially included the data of 118 individuals with an MCI diagnosis. Subsequently, we selected individuals with data on specific means of transportation in the IADL scale, which resulted in data from 117 participants being used for analysis (Figure 1).

Index for mode of transportation

To determine participants’ independence in daily modes of transportation, we used a questionnaire from the Lawton IADL scale [18], a self-report scale that provides information about the functional skills necessary to live independently in the community. The IADL assesses eight tasks: ability to use the telephone, shopping, food preparation, housekeeping, laundry, mode of transportation, responsibility for own medications, and ability to handle finances. We used the mode of transportation dimension for dividing the subjects into two groups. Participants who selected “Travels independently on public transportation or drives own car” were defined as the independent mobility group (n = 85; Figure 1). Participants who selected “Arranges own travel via taxi, but does not otherwise use public transportation,” “Travels on public transportation when assisted or accompanied by another,” or “Travel limited to taxi or automobile with assistance of another” were defined as the dependent mobility group (n = 32; Figure 1). None of the participants selected “Does not travel at all” in the IADL.

To further confirm whether participants could actually drive vehicles, we used a self-report questionnaire on the mode of transportation for long-distance travel, which was part of the living environment and health behaviors scale created for this cohort study. The options given were: (i) Driving a car or motorcycle; (ii) Riding in a family member's or friend's car or taking a taxi; (iii) Bicycle; (iv) Train or bus. Participants who responded that they “drive a private car or motorcycle” were considered to have driving ability.

Cognitive assessments

The MMSE [19] and MoCA-J [20,21] were used to assess cognitive function. The MMSE total score and its 11 subscales (orientation time, orientation place, registration, attention, recall, naming, command, repetition, read and obey, writing, and copy scores) were analyzed to explore their relationships with independence in mode of transportation. Similarly, the MoCA-J total and its seven subscales (the visuospatial executive, naming, attention, language, abstraction, delayed recall, and orientation scores) were also employed to analyze the associations with independence in mode of transportation.

Statistical analyses

Statistical analyses and data visualization were performed using Python (ver. 3.7.6), Scipy (ver. 1.7.3), and Seaborn (ver. 0.12.0). In the MCI group, the relationship between mobility independence and cognitive function was assessed using the Mann-Whitney U test for continuous values and Fisher’s exact probability test for discrete values. To further explore this relationship while accounting for potential confounding factors, an analysis of covariance (ANCOVA) was performed against the total MMSE and MoCA-J scores using age, sex, and education duration as covariates. A significance level of p < 0.05 was considered for all analyses.

## Results

Clinical and demographic characteristics

Comparisons between the independent and dependent mobility groups in clinical and demographic characteristics, as well as cognitive assessments at baseline, are summarized in Table 1. The results indicated significant differences between the groups, with a higher proportion of men, younger age, higher rate of alcohol consumption, greater driving ability, and higher prevalence of medical history of hypercholesterolemia observed in the independent mobility group.

**Table 1:** Participants’ baseline characteristics Note: MMSE, Mini-Mental State Examination; MoCA-J, Japanese version of Montreal Cognitive Assessment; independent mobility group, people who drive a car or take public transportation by themselves; dependent mobility group, people who take a taxi, public transportation with assistance, and a taxi or car with assistance; MMSE, Mini-Mental State Examination; MoCA-J, Japanese version of Montreal Cognitive Assessment. We used the Mann–Whitney U test for continuous variables, as some variables were not amenable to parametric testing, and we used Fisher’s exact test for categorical variables. ^#^ In the independent and dependent groups, 14 and 7 people, respectively, are missing driving ability data. *p < 0.05, **p < .01 in the Mann–Whitney U test. ^†^p < 0.05, ^††^p < 0.01 in the Fisher’s exact test. *** ratio of MMSE subscale score 0:1; for example, in the Independent group of Language repetition, 4:81,  4 people for 0 and 81 people for 1.

Parameters	Independent (n = 85)	Dependent (n = 32)	p value
Sex (male), n (%)	48 (56.5%)	4 (12.5%)	< 0.01^††^
Age (years), median (IQR)	75 (70–77)	80 (76.8–84.2)	< 0.01**
Education (years), median (IQR)	12.0 (9–12)	12.0 (9–12)	0.221
Body mass index (kg/m²), median (IQR)	22.9 (21.3–24.8)	23.8 (21.7–25.7)	0.627
Ever smoked, n (%)	5 (5.88%)	0 (0%)	0.321
Ever drank, n (%)	38 (44.7%)	4 (12.5%)	< 0.01^††^
Hypertension, n (%)	45 (52.9%)	21 (65.6%)	0.296
Diabetes mellitus, n (%)	20 (23.5%)	4 (12.5%)	0.304
Hypercholesterolemia, n (%)	20 (23.5%)	17 (53.1%)	< 0.01^††^
Driving ability^#^, n (%)	53 (74.6%)	3 (12.0%)	< 0.01^††^
MoCA-J total score, median (IQR)	23 (20–25)	19 (17–23)	< 0.01**
Visuospatial executive, median (IQR)	4 (3–5)	3 (2–5)	< 0.01**
Naming, median (IQR)	3 (2.25–3)	3 (2–3)	0.020*
Attention, median (IQR)	5 (4–6)	5 (4–6)	0.629
Language, median (IQR)	1 (1–2)	1 (0–2)	0.126
Abstraction, median (IQR)	2 (1–2)	2 (1–2)	0.355
Delayed recall, median (IQR)	1 (0–3)	0 (0–3)	0.138
Orientation, median (IQR)	6 (6–6)	6 (5–6)	0.011*
MMSE total score, median (IQR)	26 (25–28)	26 (24.75–28)	0.152
Orientation time, median (IQR)	5 (5–5)	5 (4–5)	< 0.01**
Orientation place, median (IQR)	5 (5–5)	4.5 (4–5)	< 0.01**
Registration, median (IQR)	3 (3–3)	3 (3–3)	0.308
Attention, median (IQR)	3 (2–5)	4 (2–4)	0.248
Recall, median (IQR)	3 (2–3)	2 (2–3)	0.257
Language naming, median (IQR)	2 (2–2)	2 (2–2)	-
Language command, median (IQR)	3 (3–3)	3 (3–3)	0.257
Language repetition, 0:1***	4:81	3:29	0.390
Language read and obey, 0:1***	2:83	0:32	-
Language writing, 0:1***	11:74	3:29	0.755
Copy, 0:1***	4:81	3:29	0.390

**Figure 2:**
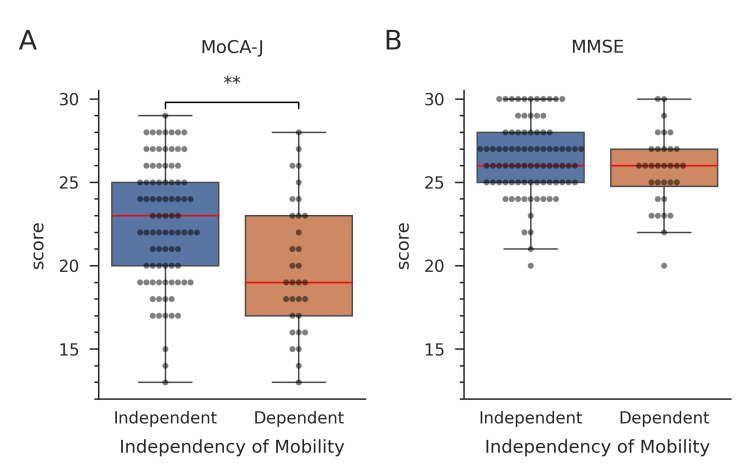
Box plots representing cognitive test scores (a) Japanese version of Montreal Cognitive Assessment (MoCA-J) and (b) Mini-Mental State Examination (MSME). Participants were divided into two groups: independent and dependent mobility. Numbers and medians of scores are shown at the bottom within the graph. Mann–Whitney U test results are shown. ** p < 0.01.

In the MoCA-J, total scores (Figure 2a), visuospatial and executive function, naming, and orientation were significantly higher in the independent mobility group compared to the dependent mobility group. Although there were no significant differences in the median scores for naming and orientation, higher first quartiles were observed for these scores. In the MMSE, scores for orientation of time and place were significantly higher in the independent mobility group compared to the dependent mobility group. Moreover, there were no significant differences in MMSE total scores between the two groups (Figure 2b).

ANCOVA on total scores of MMSE and MoCA-J

To elucidate the relationship between mobility independence and cognitive function, we conducted an ANCOVA against total MMSE and MoCA-J scores, adjusting for well-established covariates of cognitive function: age, sex, and education duration (Table 1) [22]. The results demonstrated that the MMSE and MoCA-J total scores were significantly higher in the independent mobility group compared to the dependent mobility group, after controlling for age, sex, and education. Specifically, the total MoCA-J scores demonstrated a statistically significant difference, meeting all ANCOVA assumptions, including equivariance, normality of residuals, and presence of interaction terms, with a 1.99-point higher score observed in the independent mobility group compared with the dependent mobility group.

**Table 2:** Results of the ANCOVA for total scores of MoCA-J and MMSE Least square means of each group and p-values calculated in the analysis of covariance (ANCOVA) are presented. MMSE, Mini-Mental State Examination; MoCA-J, Japanese version of Montreal Cognitive Assessment. *p < 0.05 in the ANCOVA between neuropsychological test and mobility independence (dependent: 0, independent: 1) adjusted by multiple covariates; age, sex, and education duration.

Parameters	Independent (n = 85)	Dependent (n = 32)	β (95% CI)	p value
MoCA-J mean total score	22.39	20.40	1.99 (0.223 - 3.76)	0.028*
MMSE mean total score	26.55	25.41	1.14 (0.0655 - 2.21)	0.038*

## Discussion

This study demonstrated that mobility independence was associated with cognitive function among participants. Individuals who drove themselves or independently arranged their travel using public transportation scored higher on the MMSE and MoCA-J compared to those who relied on others for transportation. Moreover, the results indicated that participants who relied on others for transportation experienced cognitive decline at the MCI stage. These findings suggest that the mode of transportation could impact cognitive maintenance during this stage. Participants who did not drive or arrange their own transportation had lower total MMSE and MoCA-J scores compared to those who maintained independence in transportation. Additionally, a significant majority (74.6%) of the independent mobility group possessed driving ability, whereas this was less common in the dependent group. Among those with driving ability in the independent group, higher MoCA-J total scores were observed compared with those without driving ability (data not shown). 

Given that Usuki city is primarily automobile-oriented with limited public transportation coverage, these results imply that preserving the ability to drive is crucial for not only daily activities but also the prevention of cognitive decline among older adults. Former drivers often exhibit lower cognitive functions compared to current drivers, and cessation of driving may contribute to cognitive impairment [13,23]. Moreover, the risk of functional limitations increases when individuals cannot move independently, affecting their ability to fulfil daily needs, such as shopping, social activities, leisure, and visiting friends [24-26]. Therefore, social interventions are essential to support the retention of driving ability. There is a higher risk of driving impairment among individuals with cognitive dysfunction compared to those without dementia [27-29]. Japan’s licensing policies for older drivers are becoming stricter than many other industrialized countries [30], resulting in licenses being revoked or suspended, for instance, in cases of drivers with dementia. In addition, there is a growing number of voluntarily surrendered licenses annually. Therefore, greater attention is needed to monitor cognitive decline progression and provide support for older individuals who have ceased driving or are unable to move independently but desire active lifestyles [31].

One conceptual model of driver behavior and corresponding cognitive abilities comprises three skill levels: strategic (planning), tactical (maneuvering), and operational (control) [32]. The strategic level involves the overall planning stages of a trip, encompassing objectives, route planning, mode selection, and assessing cost versus risk. Before movement is initiated, it is crucial to plan the destination, route, and time of departure and arrival. Therefore, orientation and executive function are critical abilities required for the strategic level of mobility. The tactical level encompasses visuospatial abilities that impact driving performance, such as obstacle avoidance, gap judgment, turning, and overtaking. Visuospatial cognitive function declines with age and is closely related to driving performance [33,34]. Cognitive domains relevant to driving performance were particularly impaired in participants who lacked independent mobility. In other words, declines in the perception of time and visuospatial abilities may be key cognitive factors predicting impaired driving among older adults with cognitive impairment. Therefore, when evaluating the driving capacity of older individuals, assessing perception of time and visuospatial abilities is particularly important. It may be worth evaluating those functions using more specified methods in future research.

This study had several limitations. First, due to its cross-sectional design, we were unable to determine the causal direction of the association between mode of transportation and cognitive function. It is possible that participants ceased driving because of perceived cognitive decline. Therefore, we believe that future research requires more detailed information collection, such as driving history and frequency, as well as prospective studies. Previous research on cognitive function and driving abilities focused on cognitive impairment as a risk factor for driving cessation [35,36]. Second, the mode of transportation was categorized solely based on the IADL in this study. Future studies using additional standardized measures of lifestyle are required to better understand the effects of transportation behaviors, such as driving or other aspects of mobility independence, on cognitive function in individuals with MCI. These studies should also explore how these effects may vary based on different transportation characteristics and physical capabilities. Third, owing to the small sample size, cognitive function was evaluated using only MMSE and MoCA-J; other stages or subtypes of MCI, such as multidomain and single-domain non-amnestic MCI [17], were not evaluated. Moreover, our sample was limited to adults with MCI aged 65 years or older. Additionally, only MoCA-J total scores met all assumptions for ANCOVA analysis. Therefore, larger sample sizes are necessary to improve the reliability of our statistical analyses. Furthermore, given that the subscales of MMSE and MoCA-J were discrete variables with narrow variation, these characteristics may not be ideally suited for ANCOVA within our limited sample size.

## Conclusions

We identified a relationship between daily modes of transportation and cognitive function in MCI patients. Specifically, participants who could move independently exhibited better cognitive functions compared to those in the dependent group. These findings may contribute to the development of new interventions for preventing the transition from MCI to dementia by further validation of causal relationships in longitudinal or intervention study.
